# Modulation of Spinal Mu-Opioid Receptor Expression by Selective Adenosine A1 and A3 Receptor Agonists and Allopurinol in a Rat Model of Neuropathic Pain

**DOI:** 10.3390/medicina62081429

**Published:** 2026-07-23

**Authors:** Jaesuk Kim, Noh Hyun Kim, Jin Deok Joo, So Young Kwon

**Affiliations:** Department of Anesthesiology and Pain Medicine, St. Vincent’s Hospital, College of Medicine, The Catholic University of Korea, Seoul 16247, Republic of Korea

**Keywords:** neuralgia, receptors, opioid, mu, receptors, adenosine, allopurinol, neuroinflammation, spinal cord, rats, Sprague-Dawley

## Abstract

*Background and Objectives:* Neuropathic pain presents a significant therapeutic challenge, often due to resistance to opioids. This study examined how selective adenosine receptor agonists and allopurinol modulate spinal mu-opioid receptor (MOR) mRNA expression in a rat model of neuropathic pain, aiming to develop a novel strategy for restoring opioid homeostasis. *Materials and Methods:* Male Sprague-Dawley rats were subjected to L5 spinal nerve ligation (SNL). The animals received treatment with selective A1AR (CCPA, 1 mg/kg) or A3AR (IB-MECA, 1 mg/kg) agonists, or allopurinol (10 or 50 mg/kg) for three days. Spinal MOR mRNA expression was measured on day 7 post-SNL using qRT-PCR. *Results:* SNL resulted in a non-significant upward trend in MOR transcription. Selective agonists (CCPA and IB-MECA) appeared to enhance or maintain MOR levels compared to the vehicle-treated group, but the most pronounced trend toward recovery of MOR mRNA expression was observed with high-dose allopurinol (50 mg/kg). Effect size analysis revealed large Cohen’s d values for high-dose allopurinol (d = 1.05) and CCPA (d = 0.90) versus sham, supporting the biological relevance of these preliminary trends. *Conclusions:* These preliminary findings suggest that augmenting endogenous adenosine bioavailability with allopurinol may be more effective than targeting individual receptor subtypes in modulating the spinal opioid system. As this is an exploratory pilot investigation, these results should be interpreted as hypothesis-generating rather than confirmatory. Since allopurinol is already a clinically established drug, these findings offer a mechanistic rationale warranting further investigation of its potential as an adjuvant for opioid resistance in chronic pain management.

## 1. Introduction

Understanding the molecular mechanisms underlying neuropathic pain is essential for developing effective therapeutic strategies in anesthesiology and pain medicine [1]. Neuropathic pain, which affects an estimated 7–10% of the global population, arises from a lesion or disease of the somatosensory nervous system and is characterized by spontaneous pain, allodynia, and hyperalgesia [2,3]. Unlike acute nociceptive pain, neuropathic pain follows a chronic course that is often refractory to conventional pharmacological treatments, imposing a substantial burden on patients and healthcare systems worldwide [2,3].

The endogenous antinociceptive system relies on descending inhibitory pathways from the periaqueductal gray and rostral ventromedial medulla that modulate nociceptive transmission at the spinal dorsal horn through opioidergic, noradrenergic, and serotonergic mechanisms. Within the opioid receptor family, the mu (MOR), delta, and kappa opioid receptor subtypes mediate distinct components of analgesia, with MOR activation providing the most potent spinal and supraspinal antinociception and remaining the principal pharmacological target for severe neuropathic pain [4]. However, current first-line pharmacotherapies for neuropathic pain—including gabapentinoids, tricyclic antidepressants, and serotonin-noradrenaline reuptake inhibitors, as well as condition-specific agents such as the sodium-channel blocker carbamazepine for trigeminal neuralgia and adjunctive atypical antipsychotics used off-label in refractory cases—provide only partial relief in a large proportion of patients and are frequently limited by dose-dependent sedation, cognitive impairment, or cardiovascular and hepatic adverse effects [5,6]. These limitations, compounded by the frequent inadequacy of opioid monotherapy itself, highlight an unmet need for adjunctive strategies that restore the function of the endogenous opioid system rather than introduce additional exogenous analgesics.

From a theoretical standpoint, restoring MOR transcriptional expression—rather than developing new opioid ligands—represents a receptor-reserve-based strategy: analgesic efficacy depends on the density of functional receptors available for agonist binding, so even a partial increase in MOR availability could lower the opioid dose required for adequate analgesia, thereby improving the therapeutic index and reducing dose-limiting adverse effects. This theoretical framework provides the rationale for investigating adenosine-receptor-targeted MOR modulation as an adjunctive, opioid-sparing strategy in the present study.

Although mu-opioid receptor (MOR) agonists represent one of the most potent analgesic options available for severe pain, their clinical utility in neuropathic conditions is frequently limited by the development of opioid resistance [2,5,7]. This resistance is increasingly understood to arise not solely from receptor desensitization or tolerance, but from active neuroinflammatory processes that impair MOR expression at the transcriptional level [5,7,8]. Specifically, peripheral nerve injury triggers the activation of spinal microglia and astrocytes, which release pro-inflammatory cytokines including tumor necrosis factor-alpha (TNF-α) and interleukin-1 beta (IL-1β) [9,10]. These mediators activate nuclear factor-kappa B (NF-κB) signaling pathways, which in turn suppress MOR gene transcription in dorsal horn neurons, perpetuating a cycle of neuroinflammation and diminished opioid responsiveness [9,11].

Adenosine, a ubiquitous endogenous purine nucleoside, exerts potent antinociceptive and anti-inflammatory effects through four G protein-coupled receptor subtypes: A1, A2A, A2B, and A3 [12,13,14]. Among these, the A1 and A3 receptor subtypes (A1AR and A3AR) have garnered particular interest in pain research owing to their Gi/o-protein coupling, which inhibits adenylyl cyclase, reduces intracellular cyclic AMP (cAMP) levels, and attenuates neuronal excitability [4,13]. The A1AR is abundantly expressed in the dorsal horn of the spinal cord, where its activation produces marked analgesic effects through membrane hyperpolarization and inhibition of excitatory neurotransmitter release [4]. The A3AR has been shown to selectively modulate persistent pain states by suppressing microglial NF-κB activation and the downstream release of pro-inflammatory cytokines, including TNF-α [15,16]. Selective A1AR and A3AR agonists have been shown to suppress the transcriptional expression of major pro-inflammatory mediators in the spinal cord [9], and since these mediators are known to negatively regulate MOR expression, the adenosine system may function as a critical homeostatic regulator of the spinal opioid system [17].

In parallel, indirect augmentation of endogenous adenosine levels represents a clinically translatable complementary approach. Allopurinol, a xanthine oxidase inhibitor widely used in the management of gout and hyperuricemia, reduces the catabolism of precursor purines, thereby increasing the bioavailability of endogenous adenosine [18]. Preclinical studies have demonstrated that allopurinol exerts antinociceptive effects that are at least partially mediated through adenosine receptor activation, with evidence specifically implicating the A1AR subtype [19]. The notable advantages of allopurinol include its well-established clinical safety profile, low cost, oral bioavailability, and decades of clinical use—attributes that make it an attractive candidate for drug repurposing as a pain adjuvant [20]. Despite these promising pharmacological properties, whether allopurinol-mediated elevation of endogenous adenosine tone can directly modulate MOR transcription at the spinal level under neuropathic conditions has not previously been investigated. (Here, “adenosine tone” refers to the ambient extracellular adenosine concentration in the spinal microenvironment, determined by the balance of adenosine production from ATP catabolism and its degradation via xanthine oxidase and adenosine deaminase.)

Our group previously demonstrated that the antinociceptive and anti-inflammatory effects of tricyclic antidepressants such as amitriptyline are mediated through A3AR activation, with subsequent inhibition of MAPK/ERK signaling and reduction in pro-inflammatory cytokine production in rat neuropathic pain models [21]. These findings established a mechanistic link between the adenosine system and spinal neuroinflammation, raising the hypothesis that targeted modulation of this system could also restore opioid receptor homeostasis. However, the specific effects of selective adenosine receptor agonists and allopurinol on the transcriptional regulation of spinal MOR under neuropathic conditions remain incompletely characterized [17].

Consequently, this study aimed to investigate and compare the effects of selective A1AR and A3AR agonists (CCPA and IB-MECA, respectively) and allopurinol on MOR mRNA expression in a rat model of L5 spinal nerve ligation (SNL)-induced neuropathic pain. By juxtaposing direct receptor activation against indirect adenosine modulation, we sought to determine whether targeting the adenosine system could maintain or restore spinal MOR expression, and to identify which pharmacological strategy may be more effective—thereby offering a novel mechanistic foundation for addressing opioid resistance in neuropathic pain. We hypothesized that both direct adenosine receptor agonism and indirect augmentation of adenosine tone would attenuate the neuroinflammation-associated suppression of spinal MOR transcription following nerve injury. The primary outcome measure was relative spinal MOR mRNA expression on day 7 post-SNL, and the specific objective was to determine whether broad elevation of endogenous adenosine tone (allopurinol) or selective receptor agonism (CCPA, IB-MECA) more effectively restores this expression.

## 2. Materials and Methods

### 2.1. Animals and Surgical Procedures

Male Sprague-Dawley rats (200–250 g, OrientBio, Seongnam, Republic of Korea) were used in this study. A total of 38 animals were included across all experimental groups. Upon arrival, animals were acclimatized for seven days prior to any experimental procedure under a temperature-controlled environment (22 ± 2 °C) with a 12 h light/dark cycle and ad libitum access to standard chow and water. Body weight was monitored throughout the experimental period. Animals were randomly assigned to their respective experimental groups using a random number table. All experimental protocols were conducted in strict accordance with the National Institutes of Health (NIH) Guide for the Care and Use of Laboratory Animals and received formal approval from the Institutional Animal Care and Use Committee (IACUC) of St. Vincent’s Hospital, The Catholic University of Korea (Approval No. 19-3).

Inclusion criteria were healthy adult male rats within the specified body-weight range with no pre-existing signs of illness or injury; a priori exclusion criteria consisted of overt post-surgical complications (e.g., wound dehiscence or motor deficit of the non-operated limb) or failure to meet the technical quality-control criteria for RNA/qRT-PCR analysis described below. Group allocation was performed using a random number table. Because of the overt nature of the surgical intervention, the surgeon performing SNL/sham surgery and drug administration could not be blinded to group assignment; however, tissue samples were coded prior to RNA extraction, and personnel performing qRT-PCR and subsequent data analysis were blinded to group identity throughout. This study was designed, conducted, and reported in accordance with the ARRIVE 2.0 guidelines [22]; the completed ARRIVE checklist is provided as a Supplementary File.

### 2.2. Spinal Nerve Ligation (SNL) Model

Neuropathic pain was induced using the L5 spinal nerve ligation model, following the method originally described by Kim and Chung [23] with minor modifications [21]. Animals were anesthetized with 2.0–2.5% isoflurane delivered via a nose cone. Following confirmation of adequate anesthesia depth by absence of the pedal withdrawal reflex, a dorsal midline skin incision was made at the lumbosacral level. The paravertebral muscles were carefully retracted to expose the L4–L5 transverse processes. The left L5 spinal nerve was identified and tightly ligated with a 6–0 silk suture, distal to the dorsal root ganglion, without damaging adjacent nerve roots. The wound was closed in layers using absorbable sutures for the muscle fascia and non-absorbable sutures for the skin. In the sham-operated group, the L5 spinal nerve was exposed and isolated using an identical surgical approach but was not ligated. Animals were monitored during recovery and housed individually following surgery. The sham procedure served as the operative control to dissociate surgery-related tissue trauma from the effects of nerve injury per se. The 7-day post-operative time point was selected based on established evidence that peak MOR transcriptional changes in the lumbar spinal cord occur within the first week following peripheral nerve injury, concurrent with the establishment of central sensitisation and neuroinflammatory activation [10,24].

### 2.3. Pharmacological Interventions

The experimental subjects were divided into the following cohorts: Sham (*n* = 4), SNL (*n* = 2), Vehicle—10% dimethyl sulfoxide (DMSO; *n* = 3), CCPA (selective A1AR agonist, 1 mg/kg; *n* = 6), IB-MECA (selective A3AR agonist, 1 mg/kg; *n* = 5), Allopurinol 10 mg/kg (*n* = 4), and Allopurinol 50 mg/kg (*n* = 4). CCPA and IB-MECA were purchased from Sigma-Aldrich (St. Louis, MO, USA) and dissolved in 10% DMSO. Allopurinol (Sigma-Aldrich) was dissolved in sterile saline. All agents were administered via intraperitoneal (i.p.) injection at a fixed volume of 200 μL once daily for three consecutive days, commencing on the day of surgery (days 0–2 post-SNL). The vehicle group received 10% DMSO (200 μL, i.p.) on the same schedule. The doses of selective adenosine receptor agonists and allopurinol were selected based on prior studies demonstrating their antinociceptive efficacy in rodent pain models [19,21]. The sham and untreated SNL groups did not receive any injection.

### 2.4. RNA Isolation and Quantitative Real-Time PCR

Seven days after the SNL procedure, animals were sacrificed by deep isoflurane anesthesia followed by decapitation. The L5 spinal cord segment was rapidly dissected on ice, snap-frozen in liquid nitrogen, and stored at −80 °C until further processing. Approximately 50–100 mg of spinal cord tissue was homogenized, and total RNA was extracted using TRIzol reagent (Invitrogen, Carlsbad, CA, USA) according to the manufacturer’s protocol. RNA concentration and purity were verified spectrophotometrically (A260/A280 ≥ 1.8). Complementary DNA (cDNA) was synthesised from 1 μg of total RNA using the RevertAid First Strand cDNA Synthesis Kit (Thermo Fisher Scientific, Waltham, MA, USA) with the following conditions: 25 °C for 5 min, 42 °C for 60 min, and 70 °C for 5 min for enzyme inactivation. SYBR Green-based real-time quantification was performed using Power SYBR Green PCR Master Mix (Applied Biosystems, Foster City, CA, USA) on a QuantStudio 3 Real-Time PCR System (Applied Biosystems). The transcriptional levels of MOR (gene symbol: Oprm1) were quantified using the 2^−ΔΔCt^ method, with β-actin (Actb) as the internal reference gene [25]. Thermal cycling conditions included an initial denaturation step at 95 °C for 10 min, followed by 40 cycles of 95 °C for 15 s and 60 °C for 1 min. Primer sequences for MOR (Oprm1) and the internal reference gene β-actin are listed in Table 1. Each reaction was performed in triplicate, and all MOR mRNA values are expressed as fold change relative to the sham group mean (set to 1.0). Prior to statistical analysis, samples with undetectable (Ct/CP > 35) or aberrant β-actin (reference gene) amplification—indicative of RNA degradation or technical failure—were excluded according to this pre-specified quality-control criterion. This resulted in exclusion of three animals (one from the IB-MECA group and two from the high-dose allopurinol group) prior to the final analysis, accounting for the group sizes reported in Table 2; no further outlier exclusion (e.g., post hoc statistical outlier testing) was performed, and all remaining values were retained.

### 2.5. Statistical Analysis

All data are presented as mean ± standard error of the mean (SEM). Differences among experimental groups were assessed using one-way analysis of variance (ANOVA), followed by Tukey’s post hoc test for multiple pairwise comparisons. A *p*-value of less than 0.05 was considered statistically significant. All statistical analyses were performed using GraphPad Prism 9.0 (GraphPad Software, San Diego, CA, USA) and SPSS version 26.0 (IBM Corp., Armonk, NY, USA). Effect sizes (Cohen’s d) were calculated for each treatment group relative to sham, and post hoc statistical power was estimated from the observed effect sizes [26], as detailed in the Discussion and Limitations. Full one-way ANOVA (F-statistic, degrees of freedom) and Tukey HSD pairwise adjusted *p*-values for all group comparisons are provided in Supplementary Table S1.

### 2.6. Use of Artificial Intelligence Tools

During the preparation of this manuscript, Claude Sonnet 4.6 (Anthropic, San Francisco, CA, USA) was used to assist with language editing, restructuring of the Discussion section, and effect size interpretation. The authors reviewed and edited all AI-generated content and take full responsibility for the accuracy and integrity of the published work.

## 3. Results

### 3.1. Changes in Spinal MOR mRNA Expression Following SNL

**Figure 1:**
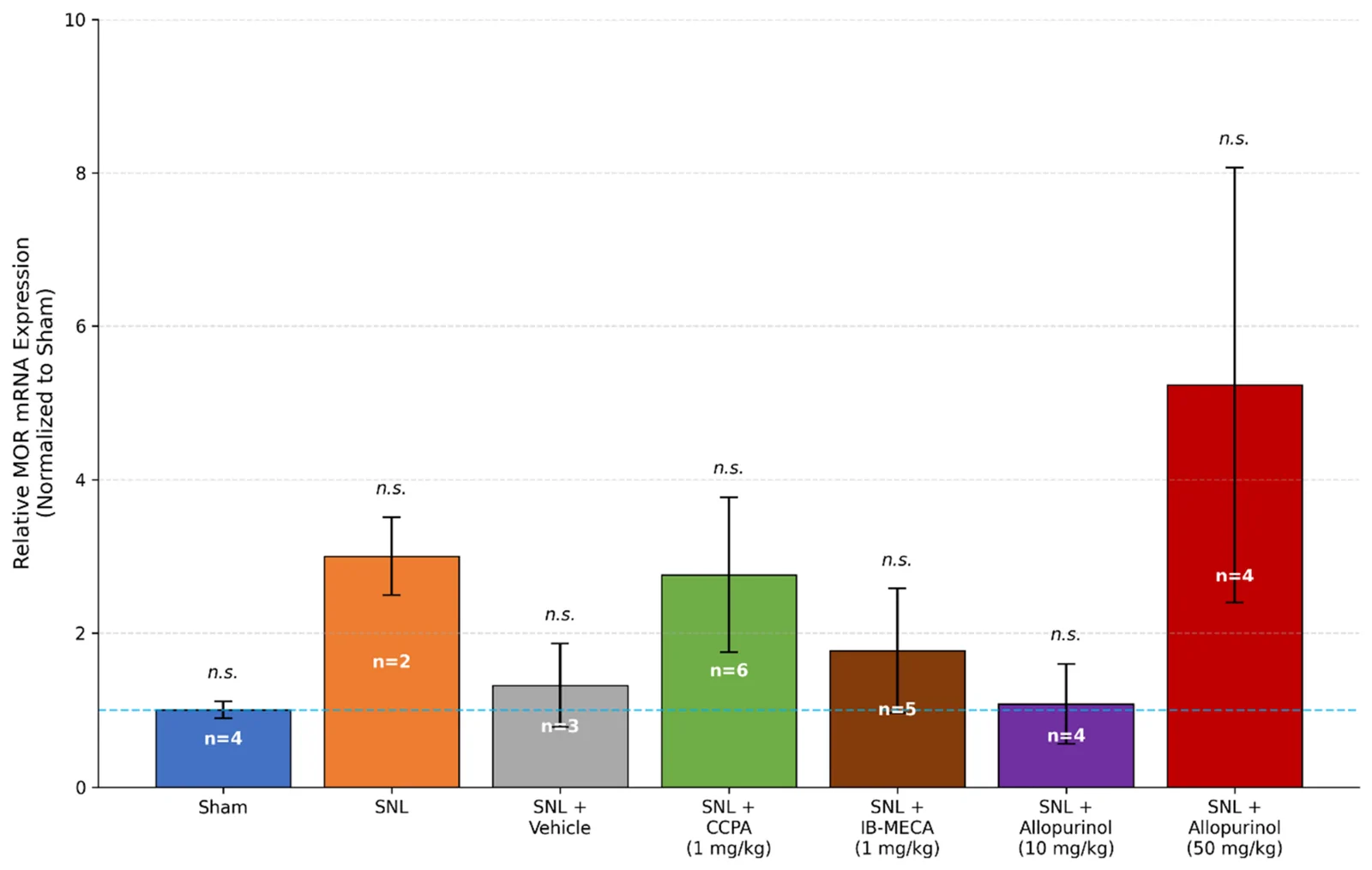
Spinal MOR mRNA expression following SNL and adenosine modulation. Spinal nerve ligation (SNL) induced a non-significant upward trend in MOR transcription. The administration of direct adenosine receptor agonists, specifically CCPA (selective A1AR agonist, 1 mg/kg) and IB-MECA (a selective A3AR agonist, 1 mg/kg), either preserved or further enhanced MOR expression compared to the vehicle-treated (10% DMSO) SNL group, although the changes were not statistically significant. Most notably, indirect modulation using allopurinol (50 mg/kg) resulted in the most pronounced recovery trend in MOR mRNA expression among all treated groups. All drugs were administered intraperitoneally once daily for three consecutive days, starting on the day of SNL. Spinal cord tissues (L5 segment) were harvested on day 7 post-SNL for qRT-PCR analysis. Data are expressed as mean ± SEM relative to the sham group (*n* = 2–6 per group). All pairwise comparisons: *p* > 0.05 (one-way ANOVA with Tukey’s post-hoc test).

To investigate the impact of neuropathic pain on the endogenous opioid system, we measured the transcriptional expression of MOR in the lumbar spinal cord seven days after L5 SNL. Results for all groups are summarized in Table 2 and illustrated in Figure 1.

In the sham-operated group, baseline MOR mRNA expression was defined as 1.00 ± 0.11 (*n* = 4). Following SNL, MOR mRNA expression showed a non-significant upward trend, reaching approximately 300% of sham levels (3.00 ± 0.51, *n* = 2; *p* > 0.05 vs. sham). Although this difference did not achieve statistical significance—likely due to the small SNL group sample size—the approximately 3-fold increase is consistent with previous reports of compensatory MOR upregulation in response to sustained nociceptive input following peripheral nerve injury [24]. This compensatory transcriptional response may reflect an attempt by the central nervous system to maintain opioid homeostasis under conditions of persistent pain signaling.

In the vehicle-treated SNL group (10% DMSO, i.p.; *n* = 3), MOR mRNA expression was 1.32 ± 0.54—lower than in the untreated SNL group (3.00 ± 0.51) but marginally elevated compared to the sham baseline. This attenuation of MOR upregulation in the DMSO group may suggest a partial confounding effect of the vehicle, though no statistically significant difference was observed (*p* > 0.05).

### 3.2. Effects of Selective Adenosine A1 and A3 Receptor Agonists on MOR Expression

Treatment with the selective A1AR agonist CCPA (1 mg/kg, i.p.; *n* = 6) resulted in a mean MOR mRNA expression of 2.76 ± 1.01 relative to sham, representing a 2.1-fold increase compared to the vehicle-treated SNL group (1.32 ± 0.54). Although this increase did not attain statistical significance (*p* > 0.05), the magnitude of MOR upregulation following CCPA treatment was notably greater than that observed in the vehicle group, suggesting a facilitatory role of A1AR activation in maintaining MOR transcription under neuroinflammatory conditions. The relatively large standard error reflects variability among individual animals, which may be attributable to differences in the degree of nerve injury, baseline expression levels, or PCR efficiency [25]. The effect size for the CCPA versus sham comparison was large (Cohen’s d = 0.90), indicating substantial biological magnitude despite the absence of statistical significance.

Administration of the selective A3AR agonist IB-MECA (1 mg/kg, i.p.; *n* = 5) resulted in a mean MOR mRNA expression of 1.77 ± 0.81 relative to sham—a moderate 1.34-fold increase compared to the vehicle group (1.32 ± 0.54). While this increase was likewise non-significant (*p* > 0.05), the directional trend is consistent with the hypothesis that A3AR signaling contributes to the maintenance of spinal opioid receptor expression by attenuating neuroinflammatory cytokine release, including TNF-α and NF-κB-mediated transcriptional suppression [9,21]. Taken together, the results from both CCPA and IB-MECA suggest a potential modulatory role of the adenosine receptor system in regulating MOR transcription, though larger studies will be required to confirm statistical significance. The corresponding effect size was medium (Cohen’s d = 0.56 vs. sham).

### 3.3. Impact of Indirect Adenosine Modulation via Allopurinol

The lower dose of allopurinol (10 mg/kg, i.p.; *n* = 4) produced a minimal effect on MOR expression (1.08 ± 0.52), nearly identical to the sham baseline, suggesting that this dose may be insufficient to generate a biologically meaningful elevation of endogenous adenosine tone in the spinal cord under neuropathic conditions [19]. Accordingly, the effect size was negligible (Cohen’s d = 0.11 vs. sham), consistent with a threshold effect below the biologically effective dose range.

In contrast, high-dose allopurinol (50 mg/kg, i.p.; *n* = 4) produced the most pronounced upward trend in MOR mRNA expression among all groups (5.23 ± 2.84)—a 5.23-fold increase relative to sham and a 3.96-fold increase compared to the vehicle group, exceeding the MOR expression levels observed in either the CCPA or IB-MECA treatment groups. Although this difference did not achieve statistical significance (*p* > 0.05 vs. all groups), the magnitude of this response is clinically meaningful and suggests that broadly increasing endogenous adenosine bioavailability may engage a wider repertoire of receptor subtypes and downstream signaling pathways than selective agonism of a single receptor [4,19]. Effect size analysis confirmed a large Cohen’s d of 1.05 versus sham, indicating substantial biological magnitude independent of statistical significance.

Collectively, these results indicate a dose-dependent pattern of MOR modulation by allopurinol and suggest that the overall “adenosine tone” within the spinal cord, rather than activation of specific receptor subtypes, may be the more critical determinant of MOR transcriptional regulation under neuropathic conditions.

## 4. Discussion

The present study investigated the effects of selective adenosine receptor agonists and allopurinol on spinal MOR mRNA expression in a rat model of L5 SNL-induced neuropathic pain. Although none of the observed changes reached statistical significance—primarily owing to small sample sizes in certain groups—consistent directional trends were observed across all adenosine-enhancing interventions, with high-dose allopurinol (50 mg/kg) producing the most pronounced upward trend (~5-fold relative to sham). These exploratory findings suggest a meaningful interaction between the adenosine system and spinal opioid receptor regulation, providing a mechanistic foundation for future investigation [19]. Importantly, effect size analysis revealed large Cohen’s d values for CCPA (d = 0.90) and high-dose allopurinol (d = 1.05) versus sham, indicating biologically substantial modulation of spinal MOR transcription. By conventional benchmarks [26], d > 0.80 constitutes a large effect, suggesting that the non-significant *p*-values reflect insufficient statistical power rather than an absence of biological effect.

A central observation of this study is the upward trend in spinal MOR mRNA expression following SNL. This pattern is consistent with prior reports of compensatory MOR upregulation in response to peripheral nerve injury, wherein sustained nociceptive input is thought to drive transcriptional activation of opioid receptor genes as part of a homeostatic response [24]. However, this compensatory upregulation appeared to be attenuated in the vehicle-treated SNL group (DMSO), suggesting that ongoing neuroinflammation may progressively impair this homeostatic mechanism. This observation reinforces the concept that the neuroinflammatory milieu plays a critical role in determining the net transcriptional output of MOR under neuropathic conditions [9,11].

Because the present study measured only MOR transcript levels, the intracellular signaling steps proposed below (e.g., PKA-dependent transcription factor phosphorylation, NF-κB nuclear translocation) were not directly assessed in this experiment; they are presented as literature-based candidate mechanisms rather than findings generated by the present data, and are offered to motivate future pathway-specific investigation.

The moderate increases in MOR mRNA expression observed following CCPA (A1AR agonist) and IB-MECA (A3AR agonist) treatment are consistent with the hypothesis that adenosine receptor activation creates a more permissive transcriptional environment for MOR expression. A1AR is a Gi/o-coupled receptor whose activation reduces intracellular cAMP levels and attenuates protein kinase A (PKA) activity, thereby modulating downstream transcription factor phosphorylation [13]. This signaling cascade may reduce the activity of transcription factors that negatively regulate MOR promoter activity under inflammatory conditions. Furthermore, A1AR activation has been shown to suppress the release of glutamate and other pro-nociceptive neurotransmitters in the dorsal horn [4], which may indirectly reduce the inflammatory signal driving MOR downregulation.

The A3AR agonist IB-MECA also produced a moderate increase in MOR mRNA expression. A3AR has been identified as a key modulator of microglial activity, with its activation shown to inhibit NF-κB signaling and suppress the production of pro-inflammatory cytokines including TNF-α and IL-1β [16]. Given that TNF-α suppresses MOR transcription through NF-κB-dependent mechanisms [9,11], A3AR-mediated cytokine suppression may preserve or partially restore MOR mRNA levels. This interpretation is supported by our group’s prior work demonstrating that A3AR activation by amitriptyline inhibits MAPK/ERK and CREB pathways while reducing pro-inflammatory cytokine production in neuropathic pain models [21].

A particularly important finding of this study is that high-dose allopurinol (50 mg/kg) produced the most pronounced upward trend in MOR expression (~5.23-fold relative to sham), exceeding the effects of selective A1AR or A3AR agonism. Allopurinol inhibits xanthine oxidase, a key enzyme in purine catabolism, thereby reducing the breakdown of hypoxanthine and xanthine and increasing the availability of endogenous adenosine through the purine salvage pathway [18,19,20]. By broadly elevating adenosine tone rather than selectively targeting a single receptor subtype, allopurinol may simultaneously engage multiple receptor-mediated signaling cascades—including A1AR-mediated neuronal hyperpolarization, A3AR-mediated cytokine suppression, and potentially A2A/A2B receptor-mediated anti-inflammatory effects [4,13]. Additionally, xanthine oxidase inhibition reduces the production of reactive oxygen species (ROS), which are themselves pro-inflammatory and may contribute to MOR downregulation through oxidative stress-dependent mechanisms [20]. The dose-dependent pattern—with 10 mg/kg showing minimal effect and 50 mg/kg demonstrating a pronounced trend—suggests a threshold effect that warrants further dose–response characterization. Mechanistically, allopurinol and its active metabolite oxypurinol inhibit xanthine oxidase, blocking the oxidation of hypoxanthine to xanthine and of xanthine to uric acid; the resulting hypoxanthine is shunted through the purine salvage pathway toward increased extracellular adenosine formation rather than being catabolized to a dead-end product [19,20]. This mechanism is consistent with recent evidence that xanthine oxidase inhibition attenuates thermal and mechanical hyperalgesia in a mouse model of peripheral mononeuropathy [27], supporting the translational consistency of allopurinol’s antinociceptive action across species and neuropathic pain models. Unlike CCPA or IB-MECA, which saturate a single receptor subtype, allopurinol’s non-selective elevation of adenosine tone may concurrently engage A1AR-mediated neuronal hyperpolarization, A3AR-mediated microglial suppression, and possibly A2A/A2B-receptor-mediated anti-inflammatory signaling, offering a plausible explanation for why high-dose allopurinol produced the largest MOR upregulation trend among all treatment arms. Direct confirmation of this multi-receptor mechanism would require receptor-subtype-selective antagonist blockade studies, which we identify as a priority for future work.

The clinical implications of these findings are considerable. Allopurinol is an inexpensive, orally bioavailable drug with a well-established safety profile that has been in clinical use for decades, primarily for the management of gout and hyperuricemia [20]. Its potential repurposing as a pain adjuvant to restore opioid receptor expression and enhance analgesic efficacy represents an attractive translational strategy that would not require development of novel compounds. Future clinical studies examining the co-administration of allopurinol with opioid analgesics in neuropathic pain patients may reveal whether the transcriptional changes observed in this preclinical model translate into improved analgesic outcomes.

Considered together, the pharmacological strategies examined here illustrate a broader trade-off in modalities for restoring MOR function in neuropathic pain. Direct opioid agonism remains the most potent analgesic approach but is constrained by tolerance, dependence, and the neuroinflammation-driven receptor downregulation motivating this study. Selective adenosine receptor agonists (CCPA, IB-MECA) offer mechanistic precision and the possibility of receptor-subtype-specific dosing, but their clinical development is comparatively early-stage and subtype-selective agonists may carry class-specific liabilities (e.g., A1AR-related bradycardia or sedation). Indirect adenosine tone augmentation with allopurinol sacrifices receptor selectivity but benefits from an established regulatory approval, decades of clinical safety data, and low cost—advantages that may outweigh its comparative lack of mechanistic precision for near-term translational purposes. Emerging modalities such as viral-vector-mediated MOR overexpression or epigenetic modulators targeting MOR promoter methylation could in principle offer more durable or cell-type-specific restoration of receptor expression, but remain considerably further from clinical application than drug-repurposing approaches such as allopurinol.

**Figure 2:**
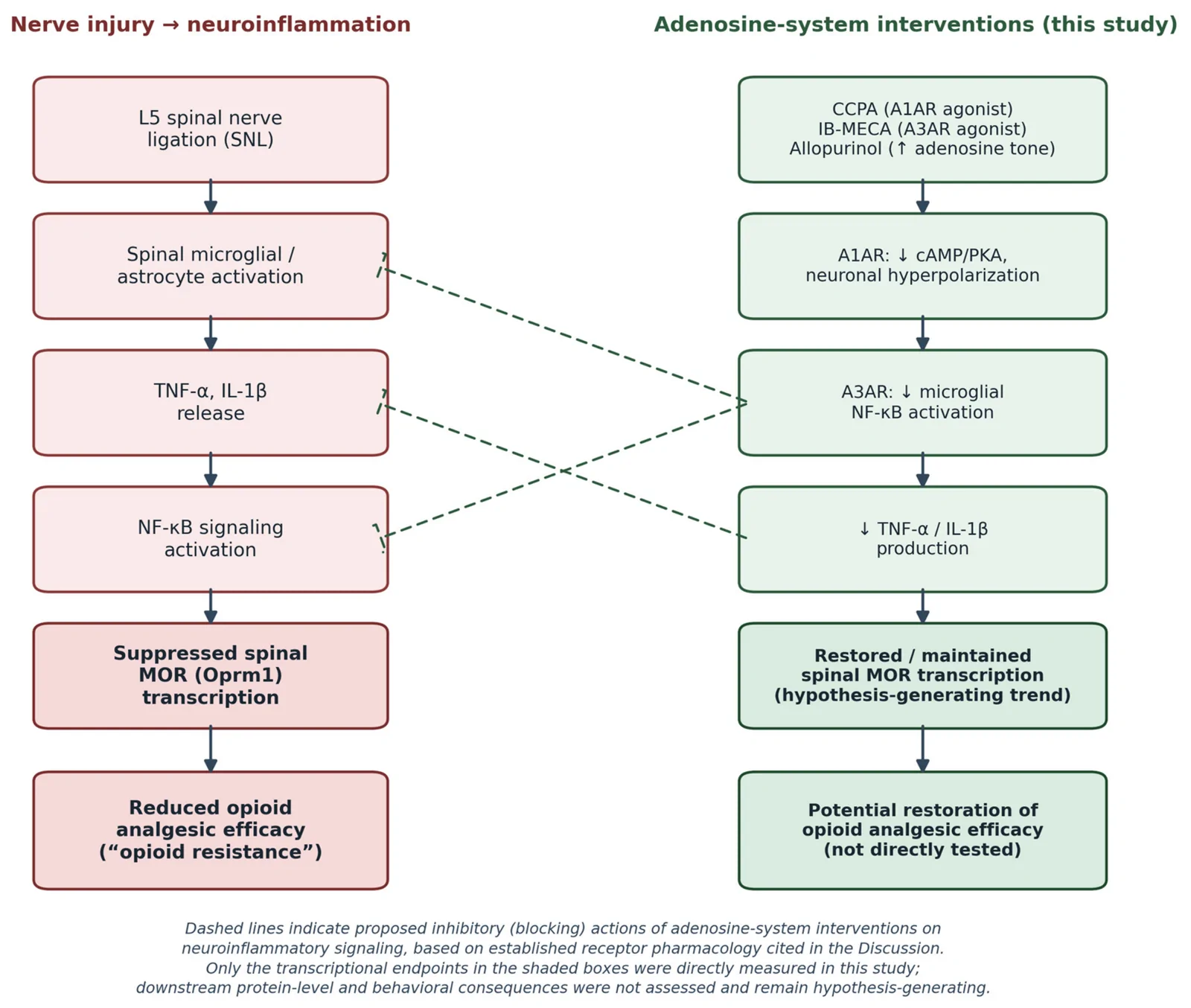
Proposed schematic pathway linking spinal nerve injury, neuroinflammatory signaling, and adenosine-system modulation of mu-opioid receptor (MOR) transcription. Nerve injury triggers spinal microglial/astrocyte activation, pro-inflammatory cytokine release (TNF-α, IL-1β), and NF-κB signaling, which suppress spinal MOR transcription and contribute to reduced opioid analgesic efficacy (**left** column). CCPA (A1AR agonist), IB-MECA (A3AR agonist), and allopurinol (indirect adenosine tone elevation) are proposed to counteract this cascade at the levels indicated (**right** column), based on established receptor pharmacology cited in the Discussion. Only the transcriptional endpoint (MOR mRNA) was directly measured in this study; all upstream and downstream steps are literature-based and hypothesis-generating. Solid arrows indicate sequential progression of pathways.

To summarize the proposed relationship between nerve injury, neuroinflammatory signaling, adenosine system modulation, and MOR-mediated antinociception, a generalized schematic pathway is provided in Figure 2.

From a translational-medicine perspective, allopurinol’s existing regulatory approval, extensive post-marketing safety record, oral bioavailability, and low cost make it well suited for rapid clinical repurposing as an opioid-sparing adjuvant, potentially bypassing much of the preclinical-to-clinical development pipeline required for a novel chemical entity. A plausible translational pathway would involve: (1) confirmatory preclinical studies incorporating protein-level and behavioral endpoints, as outlined above; (2) a phase II proof-of-concept trial evaluating allopurinol as an adjuvant to opioid therapy in patients with neuropathic pain and documented opioid hyporesponsiveness, using validated pain and opioid-sparing outcome measures; and (3) biomarker studies correlating peripheral surrogates of adenosine tone (e.g., plasma or urinary purine metabolites) with analgesic response, to support patient stratification for future trials.

Regarding dose selection, the doses employed were based on published pharmacological evidence. CCPA (1 mg/kg, i.p.) and IB-MECA (1 mg/kg, i.p.) were selected from dose ranges reported to exert antinociceptive and anti-inflammatory effects in rodent models of neuropathic pain [16,21]. The two doses of allopurinol (10 and 50 mg/kg, i.p.) were chosen to examine dose-dependency, consistent with ranges used in prior investigations of allopurinol-mediated antinociception via adenosine A1 receptor activation [19,20]. The 5-fold dose difference was intended to detect potential threshold effects in adenosine bioavailability and MOR transcriptional regulation. Although direct measurement of spinal adenosine concentrations was not performed, published evidence demonstrates that allopurinol increases extracellular adenosine levels by reducing purine catabolism through xanthine oxidase inhibition [19,20]. Future studies incorporating adenosine microdialysis or HPLC-based quantification would directly confirm this mechanism in the SNL model.

Several limitations of this study must be acknowledged. First and most importantly, the sample sizes in several groups were small, particularly the SNL group (*n* = 2), which substantially limited the statistical power to detect significant differences. Consequently, all observed effects should be interpreted as hypothesis-generating trends rather than definitive findings. Second, this study focused exclusively on mRNA expression measured by qRT-PCR. Future studies should incorporate Western blotting and immunohistochemistry to confirm whether mRNA changes translate into corresponding changes in MOR protein expression [25]. Third, behavioral outcomes—such as mechanical allodynia and thermal hyperalgesia—were not assessed in the current protocol, precluding direct correlation between MOR transcriptional changes and antinociceptive efficacy. Lastly, the mechanistic pathway linking adenosine receptor activation to MOR transcriptional regulation was not directly investigated; future studies using pathway-specific inhibitors or transcription factor assays would help delineate this mechanism. Importantly, the present study is designed and interpreted as an exploratory pilot investigation; none of the directional trends reported should be regarded as confirmatory evidence. Adequately powered, pre-registered studies with protein-level endpoints and behavioural validation are required before clinical translation can be considered. Post hoc power analysis based on the observed effect sizes indicates that approximately 15 animals per group would be required to achieve 80% statistical power (α = 0.05, two-tailed) for the high-dose allopurinol comparison (d = 1.05), and approximately 20 per group for CCPA (d = 0.90). These calculations provide a concrete quantitative basis for the design of future adequately powered studies.

Additional limitations should be noted. This study did not include a group receiving an established first-line reference analgesic for neuropathic pain (e.g., gabapentin or pregabalin), which would allow direct comparative benchmarking against standard-of-care therapy. In silico molecular docking of CCPA, IB-MECA, and allopurinol/oxypurinol against the MOR structure, and electrophysiological confirmation of antinociceptive function (e.g., dorsal horn field-potential recording or peripheral nerve conduction studies), were both beyond the scope of this transcriptional pharmacology study but represent valuable directions for future work. Finally, while personnel performing qRT-PCR and data analysis were blinded to group allocation, the surgeon performing SNL/sham surgery and drug administration could not be blinded, which is an inherent constraint of this experimental design.

Notwithstanding these limitations, the consistent directional trends observed across all adenosine-enhancing interventions provide a coherent mechanistic narrative and a compelling rationale for further investigation. Future studies employing larger sample sizes, behavioral endpoints, and protein-level analyses will be essential to validate these findings and to determine whether adenosine system modulation—particularly through allopurinol—can be leveraged as a clinically viable strategy to restore opioid receptor homeostasis in neuropathic pain [1].

## 5. Conclusions

This exploratory pilot study demonstrates that selective adenosine receptor agonists (CCPA and IB-MECA) and allopurinol exert directional trends toward maintaining or restoring spinal MOR mRNA expression in a rat model of L5 SNL-induced neuropathic pain. Although none of the observed changes reached statistical significance, effect size analysis revealed large Cohen’s d values for high-dose allopurinol (d = 1.05) and CCPA (d = 0.90), supporting the biological relevance of these preliminary findings. Notably, high-dose allopurinol (50 mg/kg) produced the most pronounced upward trend in MOR expression, suggesting that broad elevation of endogenous adenosine tone may be more effective than selective receptor agonism in modulating the spinal opioid system. Given its established clinical safety profile and availability, allopurinol represents a promising candidate for repurposing as an adjuvant strategy to address opioid resistance in chronic neuropathic pain. Adequately powered, pre-registered studies incorporating protein-level and behavioral endpoints are warranted to confirm these hypothesis-generating findings.

## Figures and Tables

**Table 1:** Primer sequences used for quantitative real-time PCR (qRT-PCR).

Gene	Sequence (5′→3′)	Accession	Product (bp)
MOR (Oprm1)	F: 5′-CTTCGGCGGAAACATCTCCA-3′ R: 5′-AGCACTGCAGCCCGTAAATC-3′	NM_012996.2	~140
β-actin	F: 5′-AGCCATGTACGTAGCCATCC-3′ R: 5′-CTCTCAGCTGTGGTGGTGAA-3′	NM_031144	228

β-actin served as the internal reference gene. MOR, mu-opioid receptor; F, forward primer; R, reverse primer.

**Table 2:** Summary of spinal MOR mRNA expression across experimental groups.

Group	*n*	Relative MOR mRNA Expression (Mean ± SEM)	Cohen’s d (vs. Sham)
Sham	4	1.00 ± 0.11	— (reference)
SNL	2	3.00 ± 0.51	4.90†
SNL + Vehicle (DMSO)	3	1.32 ± 0.54	0.52
SNL + CCPA (1 mg/kg)	6	2.76 ± 1.01	0.90
SNL + IB-MECA (1 mg/kg)	5	1.77 ± 0.81	0.56
SNL + Allopurinol (10 mg/kg)	4	1.08 ± 0.52	0.11
SNL + Allopurinol (50 mg/kg)	4	5.23 ± 2.84	1.05
		All comparisons: *p* > 0.05	† Unreliable estimate (*n* = 2)

Data expressed as mean ± SEM, normalized to the sham group. One-way ANOVA with Tukey’s post hoc test. Three animals (one from the IB-MECA group, two from the high-dose allopurinol group) were excluded prior to analysis based on the pre-specified qRT-PCR quality-control criterion described in Methods. † Unreliable estimate due to small sample size (*n* = 2).

## Data Availability

The authors confirm that the data supporting the findings of this study are available within the article.

## Supplementary Materials

The following supporting information can be downloaded at: https://www.mdpi.com/article/10.3390/medicina62081429/s1.
