# Supplementary material for: Modulation of Spinal Mu-Opioid Receptor Expression by Selective Adenosine A1 and A3 Receptor Agonists and Allopurinol in a Rat Model of Neuropathic Pain
Source: Medicina (Kaunas). 2026 Jul 23;62(8):1429. doi: 10.3390/medicina62081429 (PMC13515056; doi:10.3390/medicina62081429)
Supplement: Supplementary file 1 [file medicina-62-01429-s001.zip › medicina-4396960-supplementary.pdf]

Supplementary Table S1. Full statistical output for spinal MOR mRNA expression across experimental groups.

Group values are the per-animal MOR/β-actin CP-ratio values underlying the normalized fold-change values reported in Table 2 of the main manuscript (verified to reproduce the published group means exactly). One-way ANOVA was performed across all seven groups, followed by Tukey's HSD test for all pairwise comparisons. Three animals (one from the IB-MECA (1 mg/kg) group, two from the Allopurinol (50 mg/kg) group) were excluded prior to analysis per the pre-specified qRT-PCR quality-control criterion described in the Methods.

Overall one-way ANOVA

$F(6, 21) = 1.244$, $p = 0.325$

Group descriptive statistics

Group	n	Mean ± SEM (fold change vs. Sham)	Cohen's d vs. Sham
**Sham**	4	1.00 ± 0.11	— (reference)
**SNL**	2	3.00 ± 0.51	4.90 (unreliable, n = 2)
**SNL + Vehicle (DMSO)**	3	1.32 ± 0.54	0.52
**SNL + CCPA (1 mg/kg)**	6	2.76 ± 1.01	0.90
**SNL + IB-MECA (1 mg/kg)**	5	1.77 ± 0.81	0.56
**SNL + Allopurinol (10 mg/kg)**	4	1.08 ± 0.52	0.11
**SNL + Allopurinol (50 mg/kg)**	4	5.23 ± 2.84	1.05

Tukey HSD pairwise comparisons (adjusted p-values)

Comparison	Mean difference	Adjusted p	95% CI (lower, upper)	Significant (α=0.05)
**Allopurinol (10 mg/kg) vs Allopurinol (50 mg/kg)**	0.0096	0.341	-0.0047, 0.0239	No
**Allopurinol (10 mg/kg) vs CCPA (1**	0.0039	0.955	-0.0091, 0.0169	No

**mg/kg)**				
**Allopurinol (10 mg/kg) vs Vehicle (DMSO)**	0.0006	1.0	-0.0149, 0.0160	No
**Allopurinol (10 mg/kg) vs IB-MECA (1 mg/kg)**	0.0016	1.0	-0.0119, 0.0151	No
**Allopurinol (10 mg/kg) vs SNL**	0.0045	0.979	-0.0130, 0.0219	No
**Allopurinol (10 mg/kg) vs Sham**	-0.0002	1.0	-0.0144, 0.0141	No
**Allopurinol (50 mg/kg) vs CCPA (1 mg/kg)**	-0.0057	0.781	-0.0188, 0.0073	No
**Allopurinol (50 mg/kg) vs Vehicle (DMSO)**	-0.0091	0.496	-0.0245, 0.0064	No
**Allopurinol (50 mg/kg) vs IB-MECA (1 mg/kg)**	-0.008	0.487	-0.0216, 0.0055	No
**Allopurinol (50 mg/kg) vs SNL**	-0.0052	0.957	-0.0226, 0.0123	No
**Allopurinol (50 mg/kg) vs Sham**	-0.0098	0.321	-0.0241, 0.0045	No
**CCPA (1 mg/kg) vs Vehicle (DMSO)**	-0.0033	0.987	-0.0176, 0.0109	No
**CCPA (1 mg/kg) vs IB-MECA (1 mg/kg)**	-0.0023	0.996	-0.0145, 0.0099	No
**CCPA (1 mg/kg) vs SNL**	0.0006	1.0	-0.0159, 0.0170	No
**CCPA (1 mg/kg) vs Sham**	-0.0041	0.945	-0.0171, 0.0090	No
**Vehicle (DMSO) vs IB-MECA (1 mg/kg)**	0.001	1.0	-0.0137, 0.0158	No
**Vehicle (DMSO) vs SNL**	0.0039	0.992	-0.0145, 0.0223	No
**Vehicle**	-0.0007	1.0	-0.0162, 0.0147	No

**(DMSO) vs Sham**				
**IB-MECA (1 mg/kg) vs SNL**	0.0029	0.998	-0.0140, 0.0197	No
**IB-MECA (1 mg/kg) vs Sham**	-0.0018	0.999	-0.0153, 0.0118	No
**SNL vs Sham**	-0.0046	0.974	-0.0221, 0.0128	No

Note: Mean differences and confidence intervals are expressed in raw MOR/β-actin CP-ratio units (dimensionless); no statistically significant pairwise differences were detected at α = 0.05 for any comparison, consistent with the small per-group sample sizes discussed in the Limitations section. Cohen's d and the post-hoc power analysis reported in the main text quantify the biological magnitude of these non-significant trends independently of statistical significance.
